# CAR-T Cells: Future Perspectives

**DOI:** 10.1097/HS9.0000000000000188

**Published:** 2019-03-19

**Authors:** Sarah Charrot, Simon Hallam

**Affiliations:** 1 St. Bartholomew's Hospital, London, UK; 2 Barts Cancer Institute, QMUL, London, UK.

Chimeric antigen receptor (CAR) T cell therapy has cured cancer in some patients for whom chemotherapy had failed. The development of CAR-T cells has been a decades-long journey from when the technology was first proposed in the late 1980s to the Food and Drug Administration (FDA) approval of Novartis's tisagenlecleucel in 2017, heralding the emergence of a multibillion dollar industry. The first CAR was reported in 1993, when Eshhar et al successfully combined the cytotoxic potential of a T cell with the specific targeting of an antibody in a single gene transfer.^1^ Their first-generation CAR linked an antibody single chain variable region with either the FcRγ or CD3ζ signaling domains. While T cells transduced with this receptor demonstrated antigen-mediated activation, the earliest clinical trials with first-generation CAR-T cells in solid cancers were disappointing. Optimization of the basic construct over time has crucially included the addition of costimulatory domains leading to improved T cell activation and survival, and the identification of favorable target antigens, most prominently CD19.^2^ These developments have led to positive clinical outcomes, including reports of cure in patients with B cell malignancies.

The scope to further optimize CAR-T cell design and delivery raises the hope of a cure for many more people with malignancies, and heralds an exciting new era in cancer treatment. While researchers, physicians, patients, and investors alike are understandably seduced by such potential, there are still several major hurdles to overcome. For the vast majority of patients with blood cancer, and all with solid cancers, CAR-T cells are not yet proven to be effective, are too toxic, or are not available due to expense or geography. This perspective article aims to give an overview of where we are right now, and consider the issues that must be addressed as the field moves forward, in order to fulfill the promise of recent successes.

CAR technology is a biologically and economically powerful tool. With power comes responsibility and the European Hematology Association (EHA), along with its sibling organizations has a responsibility to realize its potential through supporting scientific progress, cooperative working across national borders and medical specialties, clinical trials, education, training and patient advocacy in government and with leaders in the biotechnology industry. In 2019, HemaSphere, the journal of the EHA, will publish a series of review articles discussing the possibilities, current problems, and future developments of CAR-T cell therapy. By the time this article is published, the EHA in conjunction with the European Society for Blood and Marrow Transplantation will have made another important step in this direction by delivering the first European CAR-T Cell Meeting in Paris. Replacing chemotherapy in the treatment of B cell malignancies will be high on the agenda, but there are even greater rewards to be gained if we are ambitious in addressing the challenges of harnessing CAR-T cells for other malignancies and ensuring global access based on need rather than ability to pay.

## The current situation

**Figure 1:**
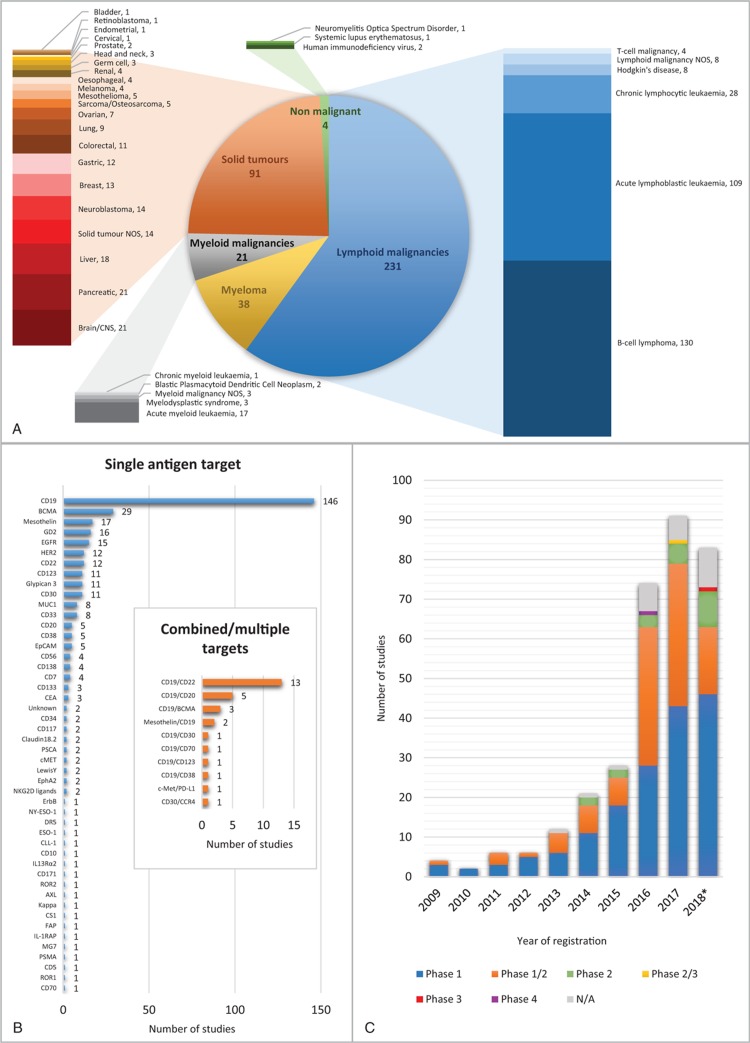
**An analysis of CAR-T trials registered at clinicaltrials.gov**. (A) Distribution of trials by condition targeted. (Note that the majority of trials target more than 1 condition; therefore, the sum does not add up to total number of trials.) (B) Distribution of trials by target antigen. Note that 12 of the trials registered do not target a specific antigen, but manufacture a personalized CAR depending on the disease phenotype. (C) The number of trials by phase and year first posted. ^∗^2018 to 7th December. CAR = chimeric antigen receptor.

**Figure 2:**
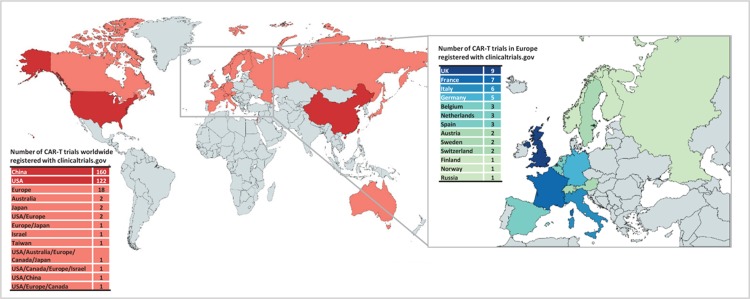
**The geographical distribution of chimeric antigen receptor-T trials registered at clinicaltrials.gov**. Map created with mapchart.net ©.

The majority of clinical trials using CAR-T cells are early phase studies in B cell malignancies. Trial activity increased dramatically in 2016 and continues at a rate of nearly 100 new trial registrations each year. The most common target is CD19, mostly alone, but increasingly in combination with other antigen targets (Fig. 1). While there is significant international co-operation, trials are overwhelmingly centered in the Northern hemisphere, with the vast majority registered in the United States and China (Fig. 2). In the United States alone, well over 1000 patients have now received CAR-T cells, and several studies have opened looking at the long-term effects in responders.

Data continue to accumulate supporting the efficacy and durability of responses to anti-CD19 CAR-T cell therapy in B cell malignancies. Response rates in acute lymphoblastic leukemia (ALL) are reported between 68% and 93%, in chronic lymphocytic leukemia between 57% and 71%, and in B cell lymphoma between 64% and 86%.^3^ Longer-term follow-up data from the ZUMA1 study indicate durable responses for the minority of patients who achieved a complete remission.^4^ Future work to better identify predictors of response will improve the risk–benefit balance and minimize unnecessary financial outlay for individual patients and healthcare systems. This could eventually result in those predicted to respond poorly to chemotherapy but well to CAR-T cells receiving them upfront.

Despite promising response rates in trials, applying this data to real-world patients is currently very challenging, partly as inclusion criteria favor better prognosis groups. Owing in part to concerns about increased rates of cytokine release syndrome (CRS) in more unwell patients with more inflammatory physiologies, combined with the risks of disease progression during the 2 to 3 weeks required for product manufacture, trials have largely excluded patients with rapidly progressive or symptomatic disease, those with poor performance status, raised inflammatory markers, fevers, cytopenias, or derangements in renal and hepatic biochemistry. Less than half of a historical cohort of chemorefractory patients with diffuse large B cell lymphoma (DLBCL) studied at 1 center would have fulfilled eligibility criteria for the ZUMA1 study. Those eligible patients had a significantly better survival with standard of care immunochemotherapy than the ineligible ones.^5^ This skewed trial subject population has created a false denominator issue, potentially overstating the efficacy and downplaying the toxicity of current products. As such, the relative magnitude of the benefit of CAR-T remains undetermined even in CD19 expressing malignancies. With future improvements in manufacture and product turnaround time, as well as refinement of CAR-T design and management of CRS, clinical trials should be better able to include more representative subjects.

Randomized studies, of which so far only 1 has opened, will better establish the place for CAR-T cells in relation to existing potentially curative therapies in B cell malignancies. The situation for other hematological malignancies and solid tumors remains a long way behind, although recent advances in myeloma and an increasing range of potential antigen targets in solid cancers have led to a large number of trials opening to address this gap.

## Future perspectives

For a variety of well-described reasons, CD19 expressing blood cancers appear most conducive to CAR-T cell therapy. High levels of tumor expression of the target antigen, ease of physical access to tumor cells through the blood and lymphatics, and the tolerability of the on-target off-tumor effect of B cell aplasia make CD19 a unique target. However, <5% of all new cancer diagnoses are CD19 expressing malignancies targetable by licensed products. The innovative strategies developed in CD19 expressing diseases to abrogate antigen-negative relapse, improve efficacy of tumor killing, improve CAR-T cell persistence, and increase control of activity and toxicity, are in parallel being pursued in efforts to bring CAR-T cell therapies to bear against other diseases. We will now look at the technical, logistic and economic challenges, ongoing innovations, and future perspectives on the journey to harness CAR-T cells against other hematological and solid malignancies. Some of the future challenges and possible solutions discussed in this section are outlined in Table 1.

**Table 1:**
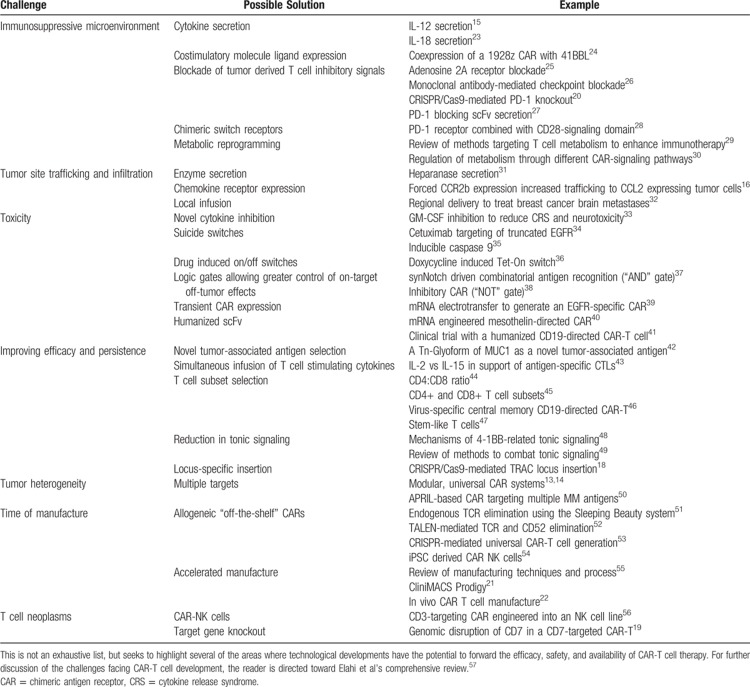
Challenges in the Future Development of CAR-T Cells

### Myeloma

Targeting the B cell maturation antigen (BCMA) in patients with myeloma is an area of great interest owing to promising early data. BCMA is expressed in nearly all cases of myeloma, and is not present on hematopoietic stem cells or nonhematological cells. Among small numbers of very heavily pretreated patients, overall response rates over 80% are being consistently reported in abstract submissions. The first published trial data of patients receiving anti-BCMA CAR-T cells reported 16 subjects with a median of 9.5 prior lines of therapy, of whom 13 responded, 11 with at least a very good partial response, with an overall median event-free survival of 31 weeks.^6^ Numbers remain too small and the follow-up too short to yet know how impactful this approach will be, but a strong proof of principle has emerged. As the technology is refined and delivered earlier in the therapeutic journey, we may see deep and durable responses that change the standard of care.

Other antigen targets are also being explored, including CD19, CD38, CD138, SLAMF7, and kappa light chains in kappa-restricted myeloma (thus sparing nonmalignant lambda-expressing plasma cells). CD138 as a target has established proof of principle through the clinical efficacy of the naked monoclonal antibody Daratumumab, but thus far progress with anti-CD138 CAR-T cells has been hampered by concerns of on-target off-tumor activity, with CD138 being expressed on erythrocytes, salivary tissue, liver, and skin. Owing to the clonal heterogeneity of myeloma, and shifting clonal tides in response to chemotherapy, it seems likely that combinatorial antigen targeting will be particularly important. Mikkilineni and Kochenderfer reviewed the progress of CAR-T cell therapy in myeloma in Blood last year.^7^

As there remain no curative chemotherapy options in myeloma despite recent progress, there is significant potential for CAR-T cells to disrupt the treatment landscape. Conversely, innovations in noncellular immunotherapies, such as antibody-drug conjugates and bispecific antibody therapies, seem set to provide stiff competition and may prove to be far less expensive.

### Myeloid malignancies

The curative potential of allogeneic hematopoietic stem cell transplantation in acute myeloid leukemia (AML) and myelodysplasia has long-established proof of concept for T cell-mediated immunotherapy in these diseases, but there are several major issues in extending this concept to the application of CAR-T cell therapy. Significant interpatient and intrapatient genetic and phenotypic heterogeneity means that there is no single AML-specific antigen to target. Furthermore, expression of AML antigens on normal healthy myeloid precursor cells risks prolonged and potentially fatal myeloablation following CAR-T cell activation. Expression of myeloid antigens on nonhematopoietic tissues risks additional on-target off-tumor effects. For example, CD33 is expressed on hepatic Kupffer cells, and hepatoxicity is a recognized side effect with the CD33 targeting antibody-drug conjugate gemtuzumab ozogomycin. The potential for long-term persistence of anti-CD33-directed CAR-T cells amplifies this risk.

There are several proposed strategies to overcome these obstacles, including (1) identification of AML-specific antigen pairings required for initiation and maintenance of leukemogenesis that can be exploited by combinatorial antigen targeting; (2) early termination of CAR-T cell activity once remission is achieved with suicide constructs or transient CAR expression techniques; and (3) myeloablative CAR-T cell therapy followed by rescue allogeneic hematopoietic stem cell transplantation. Candidate target antigens explored thus far in preclinical models include CD33, CD123, Lewis-Y, CD44v6, FLT3 receptor, CLL-1, and the folate receptor β. Several of these are being taken forward in early phase clinical trials, from which published data so far is sparse. For further insights on this field, the reader is directed toward Sarah Tasian's recent review.^8^

### T cell malignancies

Developing effective CAR-T cells against T cell malignancies, for which chemotherapy is rarely curative, will be a huge challenge. Particular, unique obstacles include contamination of the autologous CAR-T cell product by malignant T cells carrying the CAR, as well as unwanted CAR-T directed death of fellow CAR-T cells (fratricide) and of healthy T cells owing to shared target antigen. This fascinating set of challenges is reviewed by Marion Alcantara alongside Carl June and others in an elegantly succinct article.^9^ Among the proposed solutions are alternative cellular vehicles for the CAR, of which CAR-NK cells appear to be gaining most traction in early phase clinical trials against both T cell malignancies and other cancers. Further data are keenly awaited from this promising emerging field.

### Solid tumors

While immune checkpoint inhibitors have established proof of concept concerning activated T cell efficacy against solid cancers, outcomes from treating solid cancers with first-, second-, and third-generation CAR-T cell products targeting single antigens have been very disappointing. In a field with very few curative options for metastatic disease, there is clearly massive unmet clinical need, but sadly fewer patients have benefitted from CAR-T cells than review articles have been written on the subject. Among these, Long et al have produced an authoritative article outlining the particular obstacles to progress.^10^

There is a dizzying array of early phase CAR-T cell studies in solid cancers, targeting a wide variety of antigens. This reflects both the biological diversity of this field as well as the lack of persuasive data supporting any one CAR-T cell product against any particular solid tumor or tumor antigen thus far. There are some common themes that appear responsible for these disappointing results compared with blood cancers: (1) CAR-T cells face difficulty gaining access to target cells sitting within poorly vascularized tumor masses, walled-off to a certain degree by nonmalignant inflammatory cells and connective tissues. (2) On gaining access, infused CAR-T cells then face a hostile, hypoxic, and anti-inflammatory tumor microenvironment, vastly attenuating their potential cytotoxicity. (3) For those CAR-T cells that do manage to penetrate a solid tumor and retain cytotoxic potential, there is a further fundamental issue, a lack of ideal single-antigen targets. Not only is there a lack of universally expressed tumor antigens, but also a lack of specificity, with commonality of antigens on tumor and counterpart, nonredundant, healthy tissues.

Improvements to CAR-T cell design offer the potential to infiltrate and counter an immunosuppressive microenvironment, and experimental efforts are underway to increase the expression of tumor-associated antigens using radiation or epigenetic therapy with the aim of augmenting CAR-T efficacy. It may yet prove that the most realistic and effective role for CAR-T cells in solid tumors is not as a stand-alone cure, but in deepening or maintaining a chemotherapy, radiotherapy, or surgically induced remission in disease with a high risk of relapse.

It does not appear as though we are on the cusp of realizing clinical benefit with CAR-T cell therapies in solid tumors by simply repeating the techniques used in blood cancers, and it is unlikely to be a case of finding the right single-antigen target and incorporating it in to a third-generation CAR-T cell. More complex novel approaches will be needed. With improved treatment for solid cancers representing an area of such vast unmet need, and a potentially lucrative market for investors, the current drive toward tackling these issues should yield some interesting results.

### Next-generation CAR-T cells

**Figure 3:**
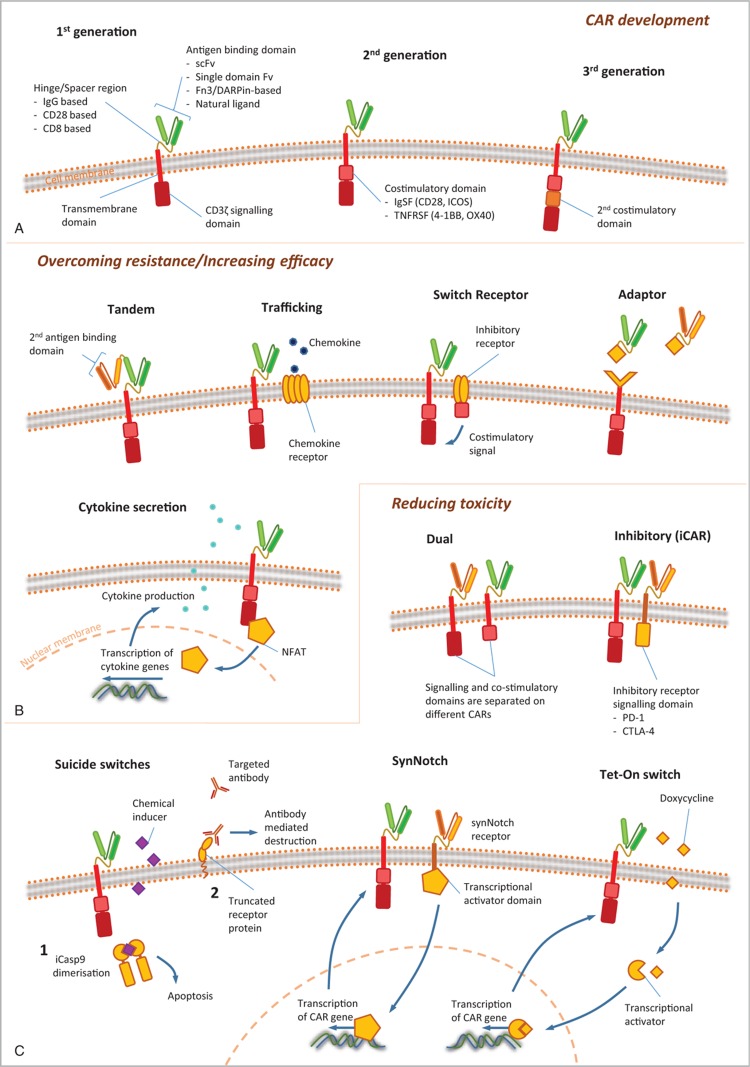
**Chimeric antigen receptor (CAR) design: novel developments in CAR technology**. (A) The CAR is made up of an antigen-binding domain, hinge or spacer region, transmembrane region, and intracellular signaling domain, ±1 or more costimulatory domains. The hinge/spacer regions and antigen-binding domains can be made from a variety of different constructs as indicated, each with their own advantages and disadvantages. Intracellular costimulatory domains, for which there are also several options, have undergone 3 generations of development, leading to improvements in CAR-T efficacy, persistence, and survival. Further structural developments seek to either improve efficacy further or to reduce toxicity. (B) Measures to improve efficacy include recognition of multiple antigens, for example tandem CARs which work through OR logic gating, allowing recognition of more than 1 antigen leading to CAR activation, and adaptor CARs which allow recognition of a common attachment, such as FITC, to which selected antigen-binding domains can be attached. Other measures are targeted at overcoming the immunosuppressive tumor microenvironment, such as switch receptors and cytokine-secreting TRUCKs. Trafficking CARs have the ability to guide themselves to the tumor site, through recognition of either chemokines or localized hypoxia, and may be further programmed to activate only in these environments to minimize on-target off-tumor toxic effects. (C) Other methods to reduce toxicity include further developments in logic gating utilizing AND (dual or SynNotch CARs) and NOT (iCARs) Boolean logic to reduce on-target off-tumor effects. CARs with integral on-off switches or suicide switches allow for rapid elimination of the CAR should toxicity prove life threatening despite maximal medical therapy. FITC = fluorescein isothiocyanate.

There have been a variety of innovations in the technical design of CAR-T cells, in an attempt to improve efficacy and reduce toxicity in hematological malignancies, and to combat the challenges of solid cancers. Figure 3 shows examples of the evolution of CAR design, with increasing complexity of structure conferring additional finesse to function. This is discussed in more detail in a recent review article published in this journal.^11^

One approach generating interest is multiple-antigen targeting, with a view to increase specificity, capture a variety of tumor clones, and reduce antigen-negative relapse. This has led to the emergence of logic gated T cells, based on the AND, OR, and NOT concepts of Boolean logic. On-target off-tumor effects can be minimized by adding AND gated circuits requiring both antigens to be present for CAR activation, or NOT gated circuits which will activate in the presence of one antigen only if the other is not present. Targeting one antigen OR another can eradicate multiple clones and reduce antigen-negative relapse, and can be achieved by either infusing 2 separate populations of CAR-T cells, transducing 2 CARs into the same cell, or by the novel tandem CAR. These principles are being investigated in clinical trials, and phase 1 data are starting to emergence on the most common combination of CD19 and CD22.^12^ The potential for further flexibility has been added with the development of adaptor-based UniCARs or ZipCARs.^13,14^ These modular receptors allow the possibility to target new antigens as they emerge, and combined with new tools for neoantigen prediction may give us the ability to stay “1-step ahead” of evolving malignancies.

Fourth-generation “armored” CARs utilize a variety of techniques to combat an immunosuppressive microenvironment. This includes cytokine secretion by TRUCKs (T cell redirected for universal cytokine killing), of which the best studied example secretes IL-12 on encountering target antigen, so shifting the tumor microenvironment in favor of immune-activation and tumor cell killing.^15^ Further developments have allowed the hostile tumor microenvironment to be utilized to direct and activate CAR-T cells, with the addition of chemokine receptors to aid trafficking, or elements to sense and activate in the presence of hypoxia.^16,17^

Augmenting the immune activating potential of CAR-T cells theoretically increases the risk of immune-mediated toxicity. This has been addressed with the addition of suicide switches, which can be triggered if required for rapid destruction of the CAR-T cell product. This principle has been extended with on-off switches, where CAR expression is dependent on ongoing drug administration, with the potential to allow external control of CAR-T cell activation, titrated to response and toxicity. The ideal might be to replicate the normal behavior of the adaptive immune system, with CAR-T cells which respond to malignant disease, effectively disappear during remission, and proliferate and activate on re-emergence of the target antigen at disease relapse.

**Figure 4:**
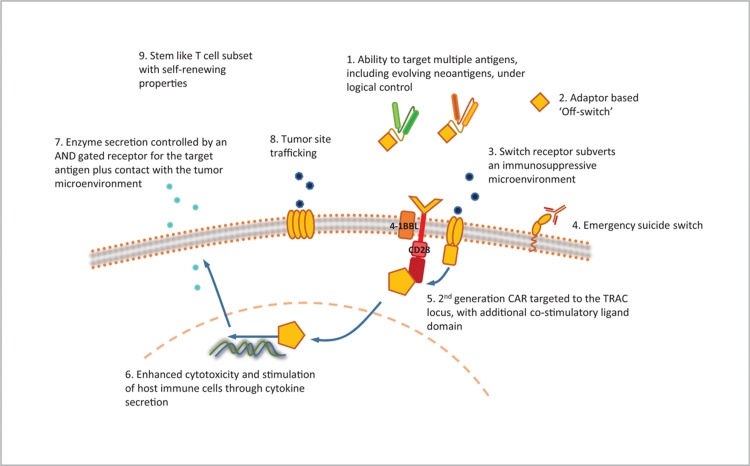
**CAR design: an idealized CAR**. With so many parallel developments advancing CAR-T technology, it will take some time to unpick the ideal methods to optimize both efficacy and safety. The “ideal CAR” will, of course, continue to change as our progress in this area continues, and we learn more about the subtleties of the technologies we have already developed. CAR = chimeric antigen receptor.

As well as changes in structural design, progress in gene editing utilizing the CRISPR/Cas9 system allows the CAR transgene to be targeted at a specific genetic locus, both increasing efficacy and reducing concerns with regard to mutagenesis.^18^ This technology also allows for “unwanted” genes to be removed, such as inhibitory signals or self-expressed target antigens (for example in T cell malignancies).^19,20^Figure 4 combines some of the features discussed above in a putative “ideal CAR” of the future.

The CAR-T cell field will not develop in isolation. It is likely that tumor responses will best be achieved and maintained by bespoke disease or patient-specific synergistic combination of CAR-T cells with other treatments such as small molecule epigenetic and immune-modulators, chemotherapy, and a host of potential antibody-based therapies. There are already several trials open which do not offer a specific CAR product, but will instead design a CAR against a number of possible antigen targets dependent on the immunophenotpying of the patient's disease. It is clear that sophisticated randomized-controlled studies will be required to identify the best way to combine this array of treatment options in the era of personalized medicine.

### CAR-T manufacture

In parallel with developments in CAR design and engineering, ongoing advances in the manufacture process will be essential to widening availability. Current centralized ex vivo processing and manufacture at often distant sites adds time, cost, and logistical complexity. Improvements in this process should expand access to patients with more rapid disease dynamics, to those living far from current centers of manufacture, and to those in less well-resourced settings. Point-of-care CAR-T cell manufacture using desktop closed-box processes building on platforms such as the Miltenyi Biotec CliniMACS Prodigy T cell Transduction Process^21^ may significantly democratize CAR-T cell production. In an ideal streamlined process, leukapheresed cells might be added to a closed-circuit machine from which multiple different characteristics of the desired CAR could be selected, with a product emerging a few days later. One step further, CAR-T cell production could conceivably occur in vivo, with a T cell-targeted transgene and transfer vehicle infused directly into the patient.^22^ In addition to the challenges of technical feasibility, this would raise major safety and control issues, but is a fascinating prospect.

Another approach to addressing this problem is the use of allogeneic CAR-T cells. Off-the-shelf products, gene edited to minimize the risks of rejection or graft versus host responses, can be rapidly administered and are also available to those unable to harvest sufficient quality or quantity of autologous cells. The scope for individualized products might be restricted, and efficacy and safety are far from established, but the speed of potential availability and the ability to batch-produce on a large scale might eventually reduce the huge financial weight attached to existing autologous products.

### Financial considerations

With 2 FDA approved products commercially available for licensed indications in ALL and DLBCL, there is a fast-growing business in delivering CAR-T cells. It is reasonable to assume that this considerable financial incentive is contributing to current hype concerning this treatment. Biotechnology companies have attracted investment in the order of billions of US dollars on the premise that CAR-T cell products will be commercially successful, but this should not distract from an objective assessment of the evidence on which we base treatment decisions. If this treatment does live up to its possibilities, then a political debate will be required concerning access to curative treatments either being a privilege for the wealthy or a right for those in need.

This is also undoubtedly a period of great opportunity. Such huge financial investment could give the scientific community huge resources to realize the potential of this emerging field, and drive forward associated discoveries in basic cancer research and immunology. While managing the balance of expectation, investment and access is not a new issue in healthcare, the unprecedented level of financial investment and the potential upheaval of the therapeutic landscape by CAR-T cells mandates vital roles for regulatory bodies, politicians, economists, and patient advocacy groups to work with biotechnology companies, researchers, and clinicians to deliver a financially sustainable CAR-T cell industry that is as equitable, safe, and effective as possible.

## Conclusions

Following almost 3 decades of development, there is now a degree of inevitably about the rise of CAR-T cell therapy. The bewildering pace of change in this field poses challenges for regulators and providers in selecting the right CAR-T products for the right patients at the right time, potentially in the right combination with other existing and emerging therapeutics. Remarkable responses in some patients with chemorefractory B cell malignancies are compelling, but are no substitute for credible long-term follow-up data from head-to-head comparisons with standard of care approaches in truly representative patient cohorts. In this respect, the added value of CAR-T therapies remains unquantified even in CD19 expressing blood cancers and their place in the treatment of other conditions is unproven and experimental. Cost and access to the 2 FDA approved products are huge issues. Growing competition and streamlining of technology and logistics should translate to more accessible pricing, although this has not always proved to be the case even with much older mainstream pharmaceuticals.

The ability to modify, enhance, and orchestrate the immune system with increasing levels of complexity to provide precise, dynamic, quasi-intelligent living therapy is far beyond that which can be achieved with conventional treatment options. Conceptually alone this justifies great hope. Our challenge now is to realize this hope for the benefit of our patients.
